# A cellular surveillance and defense system that delays aging phenotypes in *C. elegans*

**DOI:** 10.18632/aging.103134

**Published:** 2020-04-29

**Authors:** Jeong-Hoon Hahm, ChoLong Jeong, Wonhee Lee, Hee Jung Koo, Sunhee Kim, Daehee Hwang, Hong Gil Nam

**Affiliations:** 1 Center for Plant Aging Research, Institute for Basic Science, Daegu 42988, Republic of Korea; 2 Department of New Biology, DGIST, Daegu 42988, Republic of Korea; 3 Present address: Department of Biological Sciences, Seoul National University, Seoul 08826, Republic of Korea

**Keywords:** *C. elegans*, ZIP-2, aging, compensation, mitochondria

## Abstract

Physiological stresses, such as pathogen infection, are detected by “cellular Surveillance Activated Detoxification and Defenses” (cSADD) systems that trigger host defense responses. Aging is associated with physiological stress, including impaired mitochondrial function. Here, we investigated whether an endogenous cSADD pathway is activated during aging in *C. elegans*. We provide evidence that the transcription factor ZIP-2, a well-known immune response effector in *C. elegans*, is activated in response to age-associated mitochondrial dysfunction. ZIP-2 mitigates multiple aging phenotypes, including mitochondrial disintegration and reduced motility of the pharynx and intestine. Importantly, our data suggest that ZIP-2 is activated during aging independently of bacterial infection and of the transcription factors ATFS-1 and CEBP-2. Thus, ZIP-2 is a key component of an endogenous pathway that delays aging phenotypes in *C. elegans*. Our data suggest that aging coopted a compensatory strategy for regulation of aging process as a guarded process rather than a simple passive deterioration process.

## INTRODUCTION

The “cellular surveillance activated detoxification and defense” (cSADD) system in *C. elegans* maintains cellular homeostasis during physiological stress, such as pathogen infection or toxic drug treatment [1]. In cSADD, the stress-induced disruption of essential cellular activities in turn activates various protective, compensatory pathways to restore cellular homeostasis. These compensatory pathways include mitochondrial repair, drug detoxification, and immune responses. The mitochondrial unfolded protein response, which maintains mitochondrial homeostasis under stress [2], is a component of cSADD [1].

Aging, like pathogen infection, is associated with physiological stress, including the accumulation of mitochondrial damage that manifests as morphological disruption, mitochondrial DNA (mtDNA) mutation and functional decline [3–7]. Age-associated mitochondrial dysfunction is widely conserved from yeast to humans [8], and causes various age-related symptoms and diseases [9]. For instance, reduced brain function in aging is closely associated with mitochondrial dysfunction; brain mitochondria in aged animals show increased fragility, decreased rates of electron transfer, and decreased membrane potential [10]. In addition, aging is associated with decreased skeletal muscle mass and strength, decreased physical activity [11] and reduced mitochondrial density [12, 13]. Thus, mitochondrial damage is a hallmark of aging.

The nematode *C. elegans* has revealed key aging mechanisms, many of which are conserved in higher organisms [14–16]. Like humans, *C. elegans* display an age-associated loss of mitochondrial content and function [17–19]. Age-associated loss of mitochondrial integrity in the body wall muscle in *C. elegans* correlates with a decline in physical ability during aging, as assessed by measuring their maximum velocity [18].

Here, we hypothesized that aging deploys an endogenous cSADD-related pathway to maintain cellular homeostasis and prevent decline. To test this hypothesis, we screened for genes whose expression correlates with aging in *C. elegans*, and examined their capacity to protect worms from age-associated defects. We discovered that the immune response effector ZIP-2 in *C. elegans* [20] is activated during aging, and helps to maintain mitochondrial homeostasis and physical activity in aged worms. The role of ZIP-2 during aging is independent from its canonical role as an immune response effector. The age-associated cSADD-related pathway that we report will potentially inform strategies to maintain cellular homeostasis and health during aging.

## RESULTS

### Screen to uncover a cellular surveillance and defense gene that mitigates aging

To identify genes that mitigate the consequences of aging, we screened for candidates that satisfy the following criteria: their expression correlates with the degree of aging, their expression is induced in an age-dependent manner, and their disruption exacerbates aging in *C. elegans*.

To uncover candidate genes whose expression correlates with the degree of aging, we utilized our maximum velocity (MV) assay to discern physiological age. Briefly, an aging, isogenic population of *C. elegans* exhibits a heterogeneous decline in physical ability that correlates with reduced longevity [18]. To divide *C. elegans* of the same chronological age into groups of low and high physical ability, we fed worms *ad libitum* (AL), moved them to a physical assay plate with no food on day 7-8 of adulthood, and measured their MV. We defined low physical ability worms as those with an MV of less than 0.22 mm/sec, which corresponds to the minimum MV at day 1 of adulthood [18] (Supplementary Figure 1A). To uncover differentially expressed genes (DEGs) between low and high physical ability worms, we performed microarray analysis. The microarray chip contained 20,115 genes, representing most of the coding genes of *C. elegans*. We found that 10.8% of genes were differentially expressed (p < 0.05 and 1.5-fold) between low and high physical ability worms (Supplementary Table 1), including 1075 up-regulated and 1091 down-regulated genes in the low physical ability group compared to in the high physical ability group.

Enrichment analyses by Gene Ontology of Biological Processes (GOBP) and Cellular Compartments (GOCC) [21] revealed that genes involved in various regulatory processes, including transcriptional regulation (chromatin assembly, regulation of RNA metabolic process and regulation of transcription), and neuronal signaling (neurogenesis, axon guidance, and regulation of neurotransmitter levels) were up-regulated in low physical ability worms compared to high physical ability worms (Supplementary Figure 1B). In contrast, genes involved in fundamental mitochondrial functions (50 of 55 DEGs), such as electron transport chain (ETC) (*asb-1*, *atad-3*, *sdha-1*, *T20H4.5*, *ZK1128.1*), tricarboxylic acid (TCA) cycle (*sucg-1*, *idhg-2*), beta-oxidation (*acdh-1*, *acdh-13*), antioxidant defense (*sod-3*), protein quality control (*clpp-1*, *bcs-1*), and translation (*mrpl-15*, *mrpl-19*, *mrpl-20*, *mrpl-24*, *mrpl-28*, *mrps-9*, *mrps-14*, *mrps-18A*, *mrps-18C*) were down-regulated in low physical ability worms (Supplementary Figure 1C, 1D and Supplementary Table 2). These findings support our previous observations that low physical ability correlates with reduced mitochondrial function and integrity [18].

The genes that were up-regulated in low physical ability worms satisfy our first criterion, as their expression correlates with the degree of physiological aging. To identify genes that show a chronological age-dependent increase in expression, we analyzed previously reported gene expression data obtained from aging, wild-type worms [22]. We identified 1454 genes that were up-regulated during aging, and 939 genes that were down-regulated [23]. Of these, 397 and 273 genes were similarly up- or down-regulated in both the low physical ability and chronologically aged worms relative to their controls (Supplementary Figure 2A, 2B). Few genes showed discordant changes when comparing low physical ability and aged worms (Supplementary Figure 2C, 2D). Thus, low physical ability worms and chronologically aged worms display similar changes in gene expression compared to their respective controls, suggesting that lower physical ability represents a physiologically aged condition in *C. elegans* [18].

### ZIP-2, a bZIP transcription factor, delays age-associated mitochondrial dysfunction

We further investigated these candidate genes to uncover those that functionally support mitochondrial homeostasis during aging. During aging, mitochondria in the body wall muscle *C. elegans* lose their tubular morphology and gradually undergo fragmentation [18, 19]. Therefore, we examined whether any of the genes that were up-regulated in both low physical ability and aged worms were required to mitigate this age-associated mitochondrial disintegration (Supplementary Figure 3). We focused on potential master regulators, in particular 21 transcription factors (TFs) (Supplementary Table 3) with a commercially available RNAi bacterial stock.

**Figure 1:**
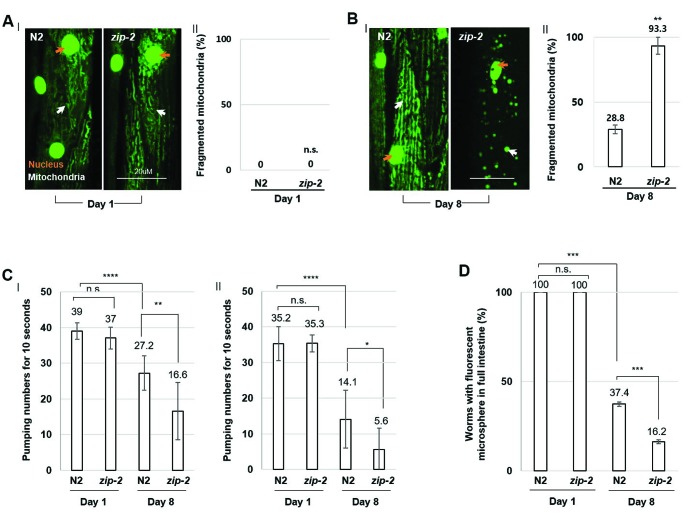
**ZIP-2 mutation accelerates *C. elegans* aging.** (**A**) (I) Representative images of mitochondrial morphologies in body wall muscle at day 1 of adulthood in wild-type (N2) (n=30) or *zip-2(ok3730)* mutant worms (n=30). The orange and white arrows indicate the nucleus and mitochondria of muscle cells, respectively. Scale bar: 20 μm. (II) Qualitative analysis of mitochondrial morphology observed at day 1 of adulthood in wild-type or *zip-2(ok3730)* mutant worms. Bars represent the proportion of worms with fragmented mitochondria. The n value represents total number of tested worms by three independent experiments. (**B**) (I) Representative images of mitochondrial morphologies in body wall muscle at day 8 of adulthood in wild-type (n=40) or *zip-2(ok3730)* mutant worms (n=35). (II) Qualitative analysis of mitochondrial morphology observed at day 8 of adulthood in wild-type or *zip-2(ok3730)* mutant worms. Bars represent the proportion of worms with fragmented mitochondria. The n value represents total number of tested worms by three independent experiments. (**C**) Pharyngeal pumping rate of 1-day wild-type (n=23), 1-day *zip-2(ok3730)* mutant worms (n=23), 8-day wild-type (n=18), and 8-day *zip-2(ok3730)* mutant worms (n=18). The n value represents total number of tested worms by two independent experiments. Error bars represent *standard deviation (S.D.).* (**D**) The proportion of worms with fluorescent microspheres in full intestine in 1-day wild-type (n=35), 1-day *zip-2(ok3730)* mutant worms (n=35), 8-day wild-type (n=54), and 8-day *zip-2(ok3730)* mutant worms (n=48). The n value represents total number of tested worms by three independent experiments. Shapiro-Wilk normality test was used to assess normal distribution of the samples. Significance was determined using a two-tailed, unpaired *t*-test. n.s.= not significant, ** P < 0.01, *** P < 0.001, **** P < 0.0001.

Of the 21 genes, we found that RNAi-mediated depletion of ZIP-2 caused a distinctive and prominent defect in mitochondrial integrity during aging. Loss of ZIP-2 increased the proportion of aged worms with fragmented mitochondria from 5% (L4440 control RNAi) to 59% (*zip-2* RNAi) (Supplementary Figure 4A). Aged mitochondria are less efficient in ATP production [17], and we found that z*ip-2* RNAi worms showed a 30% decrease in cellular ATP levels compared to control worms at day 8 of adulthood (Supplementary Figure 4B). We also analyzed the mitochondrial morphology of loss-of-function allele, *zip-2(ok3730)* mutant worms. At day 1 of adulthood, mitochondrial morphology was mostly intact in both wild-type and *zip-2(ok3730)* mutant worms (Figure 1A). However, by day 8 of adulthood, 93.3% of *zip-2(ok3730)* mutant worms had fragmented mitochondria, compared to only 28.8% of wild-type worms (Figure 1B). Thus, ZIP-2 protects mitochondrial integrity and contributes to mitochondrial function in aged worms. Overall, ZIP-2 fulfills all three criteria for an endogenous cellular surveillance and defense gene against aging.

### *zip-2* mutant worms show severe aged phenotypes

In *C. elegans*, ZIP-2 is expressed and functions in the pharynx and intestine [20, 24]. To further investigate the role of ZIP-2 in protecting *C. elegans* against aging, we monitored the age-associated decline of muscle function in pharynx or intestine by measuring the pharyngeal pumping rate or intestinal motility, respectively. Briefly, we analyzed intestinal peristalsis by feeding worms indigestible fluorescent microspheres along with heat-killed bacteria for 1 hr and measuring the accumulation of fluorescence along the intestine. As controls, we examined mutant worms with defective mitochondria, the mitochondrial electron transport chain (ETC) mutants *gas-1(fc21)* (ETC complex I) and *mev-1(kn-1)* (ETC complex II)*.* As expected, these mutants displayed a diminished pharyngeal pumping rate and lower accumulation of fluorescence along the intestine at the young adult stage (Supplementary Figure 5). Next, we examined the pharyngeal pumping rate and intestinal motility in the wild-type and *zip-2(ok3730)* mutant strain during aging. Wild-type and *zip-2(ok3730)* mutant worms had similar muscle function at day 1 of adulthood, but *zip-2(ok3730)* mutant worms had a significantly reduced pharyngeal pumping rate and intestinal motility compared to wild-type strains by day 8 of adulthood (Figure 1C, 1D). Thus, the loss of ZIP-2 accelerates the age-associated decline in muscle functions, further implying that ZIP-2 protects *C. elegans* against aging.

### ZIP-2 activity increases during aging

**Figure 2:**
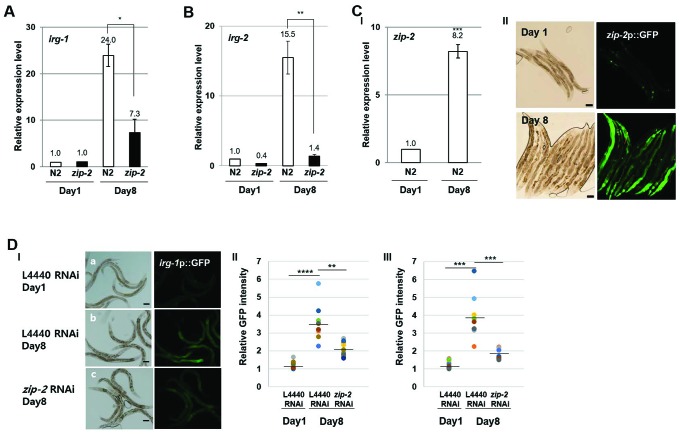
**ZIP-2 activity increases in aging.** (**A**) Relative expression levels of *irg-1* in wild-type (N2) and *zip-2(ok3730)* mutant worms at day 1 and day 8 of adulthood. (**B**) Relative expression levels of *irg-2* in wild-type and *zip-2(ok3730)* mutant worms at day 1 and day 8 of adulthood. (**C**) (I) Relative expression level of *zip-2* in wild-type at day 1 and day 8 of adulthood. (II) Representative images of *zip-2*p::GFP expression pattern at day 1 or day 8 of adulthood in wild-type strains. Scale bar: 100 μm. All relative expression levels were assessed by qRT-PCR, normalized to *act-3*. Error bars represent *SEM* by three independent experiments*.* (**D**) (I) *irg-1*p::GFP expression pattern at day 1 of adulthood in L4440 RNAi worms (a). *irg-1*p::GFP expression pattern at day 8 of adulthood in L4440 RNAi worms (b) or *zip-2* RNAi worms (c). Scale bar: 100 μm. (II and III) Relative GFP intensity in intestine. GFP intensity of individual worms was normalized to the minimum GFP intensity value among all GFP intensity values. Shown are the relative *irg-1*p*::*GFP intensities in L4440 RNAi worms (n=19) at day 1 of adulthood, in L4440 RNAi (n=19) and *zip-2* RNAi (n=20) worms at day 8 of adulthood. The n value represents total number of tested worms by two independent experiments. Shapiro-Wilk normality test was used to assess normal distribution of the samples. Significance was determined using a two-tailed, unpaired *t*-test. * P < 0.05, ** P < 0.01, *** P < 0.001, **** P < 0.0001.

To verify that a ZIP-2 pathway is induced during aging, we examined the expression of its target genes. Indeed, the expression of the ZIP-2 targets *irg-1* (Figure 2A) and *irg-2* (Figure 2B) increased 24.0-fold and 15.5-fold, respectively, from day 1 to day 8 of adulthood, and this was accompanied by a 8.2-fold increase in *zip-2* expression (Figure 2C). In contrast, *zip-2(ok3730)* mutant worms showed only a 7.3-fold increase of *irg-1* and 1.4-fold of *irg-2* from day 1 to day 8 (Figure 2A, 2B), indicating that the age-dependent increases in *irg-1* and *irg-2* expression were largely dependent on ZIP-2. We also observed increased expression of *zip-2*p::GFP and *irg-1*p::GFP transgenic reporters with aging (Figure 2C, 2D), and the promoter activity of *irg-1* was reduced in aged *zip-2* RNAi worms compared to controls (Figure 2D). These data imply that ZIP-2 is activated during aging.

### ZIP-2 activity increases in response to age-associated mitochondrial dysfunction

**Figure 3:**
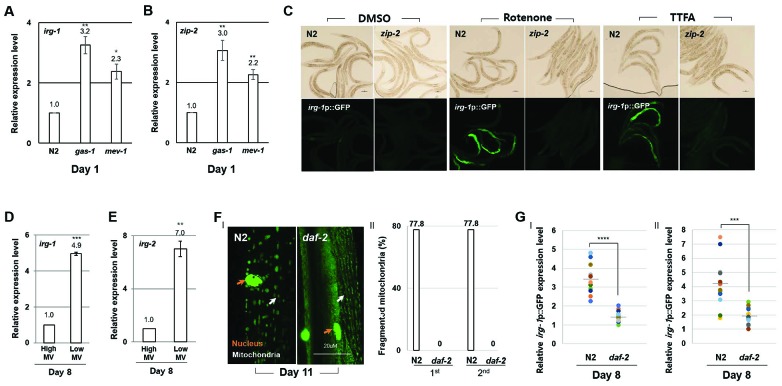
**ZIP-2 activity increases in response to mitochondrial dysfunction.** (**A**, **B**) Relative expression levels of *irg-1* (**A**) and *zip-2* (**B**) in wild-type (N2), *gas-1(fc21)* mutant, and *mev-1(kn-1)* mutant worms at day 1 of adulthood. (**C**) Representative images of *irg-1*p::GFP expression in DMSO, Rotenone (ETC complex I inhibitor) or TTFA (ETC complex II inhibitor) assay plates at day 1 of adulthood in wild-type or *zip-2(ok3730)* mutant worms. (**D**, **E**) Relative expression levels of *irg-1* (**D**) and *irg-2* (**E**) at day 8 of adulthood in worms with high MV or low MV. (**F**) (I) Representative images of mitochondrial morphologies in body wall muscle at day 11 of adulthood in wild-type strains (n=18) or *daf-2(e1370)* mutant strains (n=19). The orange and white arrows indicate the nucleus and mitochondria of muscle cells, respectively. Scale bar: 20 μm. (II) Qualitative analysis of mitochondrial morphology observed at day 11 of adulthood. Bars represent the proportion of worms with fragmented mitochondria. The n value represents total number of tested worms by two independent experiments. (**G**) Relative expression level of *irg-1*p::GFP in wild-type and *daf-2(e1370)* mutant worms at day 8 of adulthood. GFP intensity of individual worms was normalized to the minimum GFP intensity value among all GFP intensity values. Two independent experimental data. All relative expression levels were assessed by qRT-PCR, normalized to *act-3*. Error bars represent *SEM* by three independent experiments*.* Shapiro-Wilk normality test was used to assess normal distribution of the samples. Significance was determined using a two-tailed, unpaired *t*-test. * P < 0.05, ** P < 0.01, *** P < 0.001, **** P < 0.0001.

ZIP-2 has been reported to be involved in mitochondrial homeostasis and activated by mitochondrial dysfunction [24–26]. Consistent with these reports, we found that the expression of *zip-2* and of the ZIP-2 target gene *irg-1* were elevated in *gas-1(fc21)* and *mev-1(kn-1)* mutant strains compared to wild-type (Figure 3A, 3B). Further, we confirmed that treating worms with ETC inhibitors led to increased expression of the *irg-1*p::GFP reporter (Figure 3C). In contrast, treating *zip-2(ok3730)* mutant strains with ETC inhibitors did not affect the expression of *irg-1*p::GFP (Figure 3C). Importantly, we found that the expression of both *irg-1* and *irg-2* were higher in low MV worms compared to high MV worms of the same chronological age (Figure 3D, 3E). Moreover, mitochondrial damage and the activity of the *irg-1*p::GFP reporter were elevated in wild-type strains compared to long-lived *daf-2(e1370)* mutant strains at day 11 (Figure 3F, 3G) [27, 28].These data support the idea that the age-associated mitochondrial dysfunction is sufficient to induce ZIP-2 activity.

### Aging and pathogen infection activate ZIP-2 through distinct mechanisms

**Figure 4:**
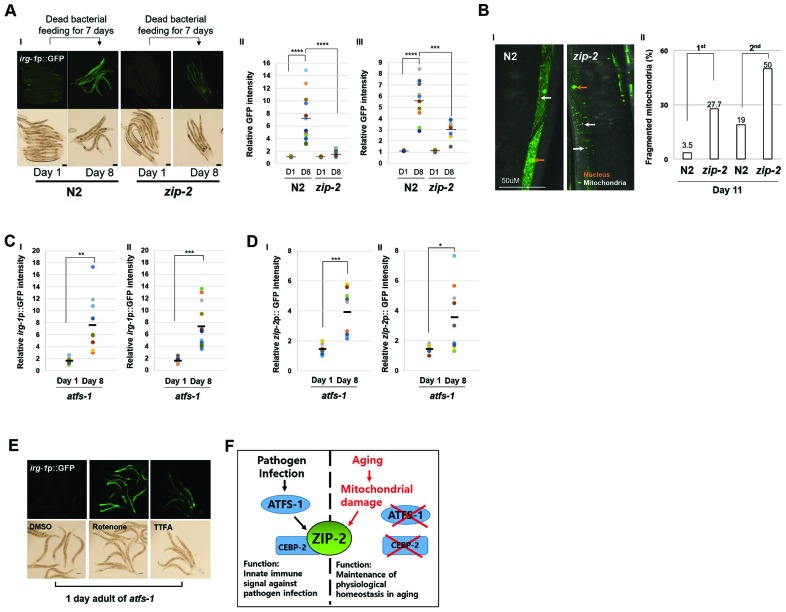
**Aging and Pathogen Infection activate ZIP-2 through a distinct mechanism.** (**A**) (I) *irg-1*p::GFP expression patterns of wild-type (N2) and *zip-2(ok3730)* mutant worms at day 1 or day 8 of adulthood. Each strain was transferred to dead bacterial plates at day 1 of adulthood. Scale bar: 100 μm. (II and III) Relative GFP intensity in intestine. D1 and D8 represent day 1 and day 8, respectively. GFP intensity of individual worms was normalized to the minimum GFP intensity value among all GFP intensity values. Two independent experimental data. (**B**) (I) Representative images of mitochondrial morphologies in body wall muscle at day 11 of adulthood in wild-type (n=49) and *zip-2(ok3730)* mutant worms (n=36). Scale bar: 50 μm. (II) Qualitative analysis of mitochondrial morphology observed at day 11 of adulthood in wild-type and *zip-2(ok3730)* mutant worms. Bars represent the proportion of worms with fragmented mitochondrial form. The n value represents total number of tested worms by two independent experiments. (**C**, **D**) Relative *irg-1*p::GFP (**C**) or *zip-2*p::GFP (**D**) intensity in *atfs-1(gk3094)* mutant worms at day 1 or day 8 of adulthood. Two independent experimental data. (**E**) *irg-1*p::GFP expression pattern at day 1 of adulthood of *atfs-1(gk3094)* in DMSO or Rotenone or TTFA assay plates. Scale bar: 100 μm. (**F**) A schematic diagram of ZIP-2 activation in aging or by pathogen infection and its biological functions in *C. elegans*. Shapiro-Wilk normality test was used to assess normal distribution of the samples. Significance was determined using a two-tailed, unpaired *t*-test. ** P < 0.01, *** P < 0.001, **** P < 0.0001.

ZIP-2 is well-known innate immune signal in *C. elegans* that is activated by pathogen infection [20]. To determine if ZIP-2 activity increases during aging due to a potential OP50 infection, we examined aged worms fed heat-killed, rather than live, OP50. Wild-type strains fed dead OP50 for 7 days still displayed increased *irg-1* promoter activity relative to *zip-2(ok3730)* mutant strains (Figure 4A). In addition, aged worms fed dead bacteria still displayed an increased proportion of worms with fragmented mitochondria upon loss of *zip-2* (Figure 4B). Overall, these data suggest that the protective role of ZIP-2 during aging does not reflect a putative OP50 infection.

CEBP-2, the *C. elegans* ortholog of human CEBPG (CCAAT enhancer binding protein gamma) (https://wormbase.org//#012-34-5), acts together with ZIP-2 to promote survival upon *P. aeruginosa* infection [26]. To test whether CEBP-2 also participates in protecting *C. elegans* against aging, we compared the mitochondrial morphology of worms fed L4440 RNAi or *cebp-2* RNAi. At day 8 of adulthood, we observed fragmented mitochondria in 33.3% of L4440 RNAi bacteria fed worms and in 22.9% of *cebp-2* RNAi bacteria fed worms (Supplementary Figure 6). Thus, CEBP-2 is not involved in mitochondrial protection during aging and ZIP-2 may not act with CEBP-2 to protect *C. elegans* against aging phenotypes.

Further, mitochondrial stress lead to nuclear localization of the transcription factor ATFS-1, which activates the mitochondrial unfolded protein response [29]. Pathogen infection is also known to trigger mitochondrial stress, re-localization of ATFS-1 and activation of ZIP-2 [25]. ZIP-2 activation by pathogen infection was partially dependent on ATFS-1, therefore we investigated whether ATFS-1 is required to activate ZIP-2 during aging. We found that *irg-1* and *zip-2* expression levels were still significantly increased during aging in *atfs-1*(*gk3094*) mutant strains (Figure 4C, 4D). Moreover, *atfs-1*(*gk3094*) mutants treated with the mitochondrial ETC inhibitors rotenone or thenoyltrifluoroacetone (TTFA) still displayed increased expression of the *irg-1*p::GFP reporter (Figure 4E). These data imply that mitochondrial damage and aging can induce ZIP-2 independently of ATFS-1. We infer that aging and pathogen infection activate ZIP-2 through distinct mechanisms (Figure 4F).

## DISCUSSION

Our work shows that *C. elegans* deploys a cellular surveillance and defense signal against aging: ZIP-2 surveils aging with mitochondrial dysfunction and mitigates age-associated physiological dysfunction. The presence of a cellular protective system during aging implies that, at least in *C. elegans*, aging is a guarded process rather than a passive deterioration process. Note that, at the same chronological age, low MV worms had more severe mitochondrial damage and higher expression levels of *irg-1*, *irg-2*, and *zip-2* than high MV worms, suggesting that there is a threshold for ZIP-2-mediated *C. elegans* protection in the aging process.

It is notable that the percentage of overlapping genes between the differentially expressed genes in worms with different physical abilities and with different chronological ages is ~27% (Supplementary Figure 2). Our data indicates that, although loss of physical ability is a hallmark of aging, it embraces a fraction of chronological aging.

ZIP-2 is well known as an innate immune signal that protects *C. elegans* against pathogen infection [20, 24]. Here, we found that ZIP-2 activity increases during aging independently of infection and of the master regulator ATFS-1 [25]. Further, CEBP-2, which partners with ZIP-2 to enhance *C. elegans* survival during pathogen infection [26], does not participate in mitochondrial protection during aging, suggesting that ZIP-2 functions differently during aging and pathogen infection. Thus, our work provides new insight into the physiological relevance of ZIP-2.

ZIP-2 is expressed and functions in the intestine (Figure 2C, 2D), yet is necessary to mitigate mitochondrial disintegration in muscle cells during aging. These data imply an indirect mechanism. We propose that ZIP-2 in intestine activates the expression protein that acts in a non-autonomous manner (e.g. [30]) or that ZIP-2 delays aging *C. elegans*, which as a consequence delays mitochondrial disintegration during aging.

We recently reported that ZIP-2 is a mediator of the dietary restriction effects in *C. elegans* [31]. Our current results further show that aging induces endogenous protective responses, via ZIP-2, that help to stabilize physiological homeostasis during the age-associated decline of cellular activities. Thus, ZIP-2 and the age-induced cellular protective systems play important roles in modulating the aging process, and may hold strategies to enhance health span.

## MATERIALS AND METHODS

### Strains

All strains were maintained at 20°C. The following strains were used in this study. N2 wild-type, *zip-2(ok3730)*, *gas-1(fc21)*, *mev-1(kn-1)*, AU133 agIs17 [*myo-2*p::mCherry + *irg-1*p::GFP] IV, PD4251 ccIs4251 [(pSAK2) *myo-3*p::GFP::LacZ::NLS + (pSAK4) *myo-3*p::mitochondrial GFP + *dpy-20*(+)]; *dyp-20(e1282)*, ERT20 jyEx6 [zip-2p::GFP + myo-2p::mCherry], PE255 feIs5 [*sur-5*p::luciferase::GFP + *rol-6(su1006)*], *zip-2(ok3730)*; ccIs4251 [(pSAK2) myo-3p::GFP:: LacZ::NLS + (pSAK4) myo-3p::mitochondrial GFP + dpy-20(+)], *zip-2(ok3730)*; agIs17 [myo-2p::mCherry + irg-1p::GFP] IV, ZC376.7(gk3094); agIs17 [myo-2p::mCherry + irg-1p::GFP] IV, ZC376.7(gk3094); jyEx6 [zip-2::GFP + myo-2p::mCherry], ZC376.7 (gk3094); agIs17 [myo-2p::mCherry + irg-1p::GFP] IV, *daf-2(e1370)*; agIs17 [myo-2p::mCherry + irg-1p::GFP] IV, and *daf-2(e1370)*; ccIs4251 [(pSAK2) myo-3p::GFP::LacZ::NLS + (pSAK4) myo-3p::mitochondrial GFP + dpy-20(+)], VC3201(*atfs-1*(*gk3094*)).

### Microarray and data analysis

The high MV or low MV worms were divided and harvested as described in Supplementary Figure 1A. Total RNA was extracted by using miRNeasy mini kit (Qiagen, Cat No. 217004) and was quantified using NanoDrop 2000 (Thermo Fisher Scientific, NanoDrop Products, Wilmington, DE, USA). RNA integrity was assessed using Agilent Bioanalyzer 2100 (Agilent Technologies, CA, USA). The RNA integrity value of all samples is greater than 9. Sample labeling, microarray hybridization, and washing were performed according to the standard protocols of the manufacturer. The total RNA was transcribed to double-stranded complementary DNA (cDNA), synthesized into complementary RNA (cRNA), and finally labeled with cyanine-3-CTP, and then hybridized onto the Agilent C. elegans (V2) Gene Expression Microarray, 4x44K microarray containing 43,803 probes. After washing, the arrays were scanned using an Agilent Surescan microarray scanner (Agilent Technologies). Feature Extraction software (version 11.5.1.1; Agilent Technologies) was used to analyze the array images and obtain raw data.

Microarray expression data that reported in this paper have been deposited in the NCBI Gene Expression Omnibus (GEO) under accession number GSE99020. https://www.ncbi.nlm.nih.gov/geo/query/acc.cgi?acc=GSE99020.

### Statistical analysis of gene expression data

To identify genes that are differentially expressed between high MV and low MV groups, the microarray data were analyzed with following procedure. The log2 value of the measured probe intensities from each microarray dataset was normalized using quantile normalization method [32]. Next, a Gaussian mixture model was applied to determine present probes. Two Gaussian probability density functions are fitted to the distribution of normalized intensities in each sample and the probes with higher intensities than a cutoff, where two Gaussian probability density functions meet, were determined to be present. Then, an integrative statistical hypothesis testing, which was previously reported [33] was conducted to the normalized intensities of each data set. A Student t test and log2 median ratio test were applied to calculate T values and log2 median ratios. Empirical null distribution for T values and log2 median ratios or each data set were estimated using 1000 permutations of samples by Gaussian kernel density estimation method. Adjusted p-values of each gene for the two tests were achieved by two-tailed tests with their corresponding empirical distributions and then two adjusted p-values were merged into combined p-values using Stouffer’s method [34]. Differentially expressed genes (DEGs) of each microarray data set were determined by three criteria (I) present probe; (II) combined p-values (<0.05); and (III) absolute log2 fold-changes (>0.58, 1.5 fold change). To identify age-associated DEGs, we re-analyzed a published time series transcriptome data (GEO accession ID: GSE12290) with previously reported method [23]. The public time series transcriptome data provides less normalized log2 ratio as an expression level of transcripts and median value of expression level for each time point is calculated to obtain median profile from day 4 of adulthood to day 24 of adulthood. To determine DEGs, the difference between log2 value of expression level at day 4 of adulthood and other time points were calculated. The genes with maximum absolute fold-change>2.5 were considered to be differentially expressed. Note that fold-change cutoff as 2.5 is determined by 95 percentile of empirical fold-change distribution which is obtained by random permutations. To address the age-dependent pattern of DEGs, expression patterns were clustered using k-means clustering method (distance measure=correlation, k=40). 40 clusters of DEGs were then classified into ‘up’, ‘down’ and ‘others’ groups using hierarchical clustering method (linkage=complete, distance metric=Euclidean).

### Enrichment analysis of gene ontology

All Gene Ontology Biological Processes (GOBPs) or Cellular Processes (GOCCs) enrichment analysis were performed using DAVID online software [21]. For each GOBP/GOCC, p-value that indicates the significance of enrichment by the group of genes was calculated. GOBPs/GOCCs with p-value<0.05 in at least one group of genes were represented.

### Measurement of worm’s maximum velocity (MV)

MV measurement was performed as previously described [18]. Single worms were transferred to the physical assay plate (NGM with no bacterial lawn) and movements recorded immediately. After recording, the worm was transferred to a fresh NGM plate. The recording period was 30 seconds at a rate of 30 frames per second. The assay conditions were as follows: 20°C and ~40% humidity, with no lid. The recording system comprised a stereomicroscope (Nikon SMZ 745T), a CCD camera (TUCSEN TCH-5.0), and imaging software (TUCSEN ISCapture). Recorded images were analyzed by ImageJ and wrMTrck (plugin for ImageJ: http://www.phage.dk/plugins/). The locomotion velocity data were imported into an Excel spreadsheet. The peak locomotion velocity observed in the 30 second period was used as the MV.

### Qualitative analysis of mitochondrial morphology

Morphological categories were defined as previously described [18]. Mitochondrial images showing a majority of long interconnected mitochondrial networks were classified as tubular, and mitochondrial images showing a majority of short mitochondria were classified as fragmented. Worms were immobilized using 100 mM sodium azide during imaging. For imaging, a microscope equipped with a C-Apochromat 40x/1.20W Korr FCS M27 and a photo-multiplier tube (PMT) are used. Zen 2011software (black edition) was used to acquire fluorescent z stacks of worms (1 μm/slice).

### RNAi experiments

For RNAi experiments, we used the commercial *C. elegans* RNAi feeding libraries generated by Ahringer laboratory (Geneservice Ltd., Cambridge, UK). RNA interference *Escherichia coli* strains were cultured as previously described [35].

### ATP measurement

The amount of ATP production was measured *in vivo* used PE255 strains as described previously [36]. Fluorescence and bioluminescence were measured using a synergy HTX multi-mode reader (BioTek). ATP level was determined by dividing bioluminescence by the respective GFP reading.

### Quantitative-RT PCR

Total RNA was extracted by using miRNeasy mini kit (Qiagen, Cat No. 217004). cDNA was generated by using a reverse transcription system (ImProm-II, Promega, Cat No. A3800) and was used for quantitative PCR. Quantitative real time PCR was performed with SYBR green dye (TOPreal™ qPCR 2X PreMIX, Enzynomics, Cat No. RT500) using CFX96^TM^ Real-time C1000 Touch Thermal cycler (Bio-Rad) and analyzed using ΔΔCt methods described in the manufacturer’s manual. Sequences of primers used for quantitative RT-PCR analysis; *zip-2*-Forward: GTTCTTTCCACAGCTTGTGC, *zip-2*-Reverse: GATGACGAATCGGACGATAC, *irg-1*-Forward: GCTGAAATTCACTTGTAGTGAG, *irg-1*-Reverse: GAGACCATAATTTCAATTGCTC, *irg-2*-Forward: CACCTCATTATTGCATTGTTTC, *irg-2*-Reverse: GTTGTAGACTTTTGAAAGGTTG, *act-3*-Forward: AAGTCATCACCGTCGGAAAC, and *act-3*-Reverse: TTCCTGGGTACATGGTGGTT.

### Assay for accumulation of fluorescent microsphere in intestine

Worms fed Fluoresbrite® YG Carboxylate Microspheres 0.50 μm diameters with heat-killed bacteria for a 1 hour and then the accumulation of fluorescent bead in the intestine was observed using an Eclipse Ni (Nikon).

### Pharyngeal pumping test

The number of contractions in the terminal bulb of pharynx was counted for 10 seconds. The pharyngeal pumping was observed by a SMZ 745T microscope (Nikon) equipped with a 2X lens (G-AL 2x, Nikon).

### Mitochondrial inhibitor test

The concentration of mitochondrial inhibitors used for testing was defined as described previously [37]. Mitochondrial inhibitors were dissolved in DMSO. The final concentration of DMSO for testing was 1% in each experimental condition. For imaging, young adult worms were treated with mitochondrial inhibitors or DMSO were for 6 hr.

## Supplementary Material

Supplementary Figures

Supplementary Table 1

Supplementary Tables 2, 3
