# Supplementary material for: A cellular surveillance and defense system that delays aging phenotypes in C. elegans
Source: Aging (Albany NY). 2020 Apr 29;12(9):8202–20. doi: 10.18632/aging.103134 (PMC7244029; doi:10.18632/aging.103134)
Supplement: Supplementary Figures [file aging-12-103134-s003..pdf]

SUPPLEMENTARY FIGURES

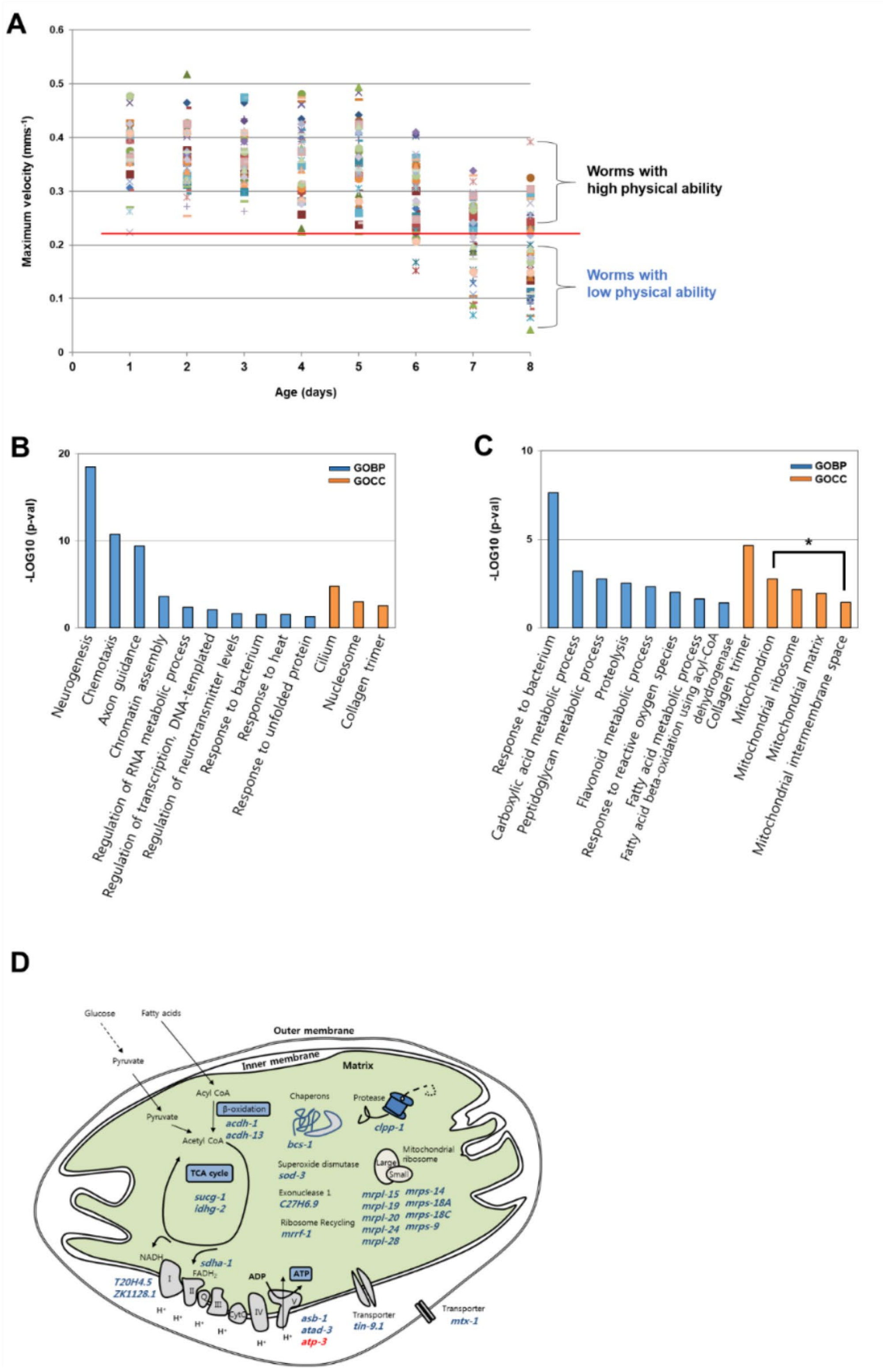

Supplementary Figure 1. Differential gene expression between worms with high MV and low MV. (**A**) Changes of maximum velocity (MV) in *C. elegans* during aging. Each notation indicates a longitudinal change of individual worms. MV is used as a criterion for physical ability and the worms were grouped into high and low physical ability groups according to their MVs at day 7 and day 8 of adulthood.

MV was measured as described previously, and groups were based on the lowest MV (0.22 mm/sec) of day 1 of adulthood [1]. (**B**) Enriched GOBP and GOCC terms of up-regulated genes in low vs. high physical ability worms. (**C**) Enriched GOBP and GOCC terms of down-regulated genes in low vs. high physical ability worms. The asterisk indicates the GOCC terms of genes related to mitochondria. (**D**) Blue letters note genes that are down-regulated in worms with low MV. These include genes that encode electron transport chain (ETC) components (*asb-1*, *atad-3*, *sdha-1*, *T20H4.5*, *ZK1128.1*), tricarboxylic acid (TCA) cycle (*sucg-1*, *idhg-2*), beta-oxidation pathway (*acdh-1*, *acdh-13*), chaperon (*bcs-1*), superoxide dismutase (*sod-3*), protease (*clpp-1*), ribosome subunits (*mrpl-15*, *mrpl-19*, *mrpl-20*, *mrpl-24*, *mrpl-28*, *mrps-9*, *mrps-14*, *mrps-18A*, *mrps-18C*), and transporters (*tin-9.1*, *mtx-1*). *atp-3* noted in red is up-regulated in worms with low MV and encodes a subunit of mitochondrial ATP synthase (complex V).

1. Hahm JH, Kim S, DiLoreto R, Shi C, Lee SJ, Murphy CT, Nam HG. C. elegans maximum velocity correlates with healthspan and is maintained in worms with an insulin receptor mutation. Nat Commun. 2015; 6:8919. https://doi.org/10.1038/ncomms9919 PMID:26586186

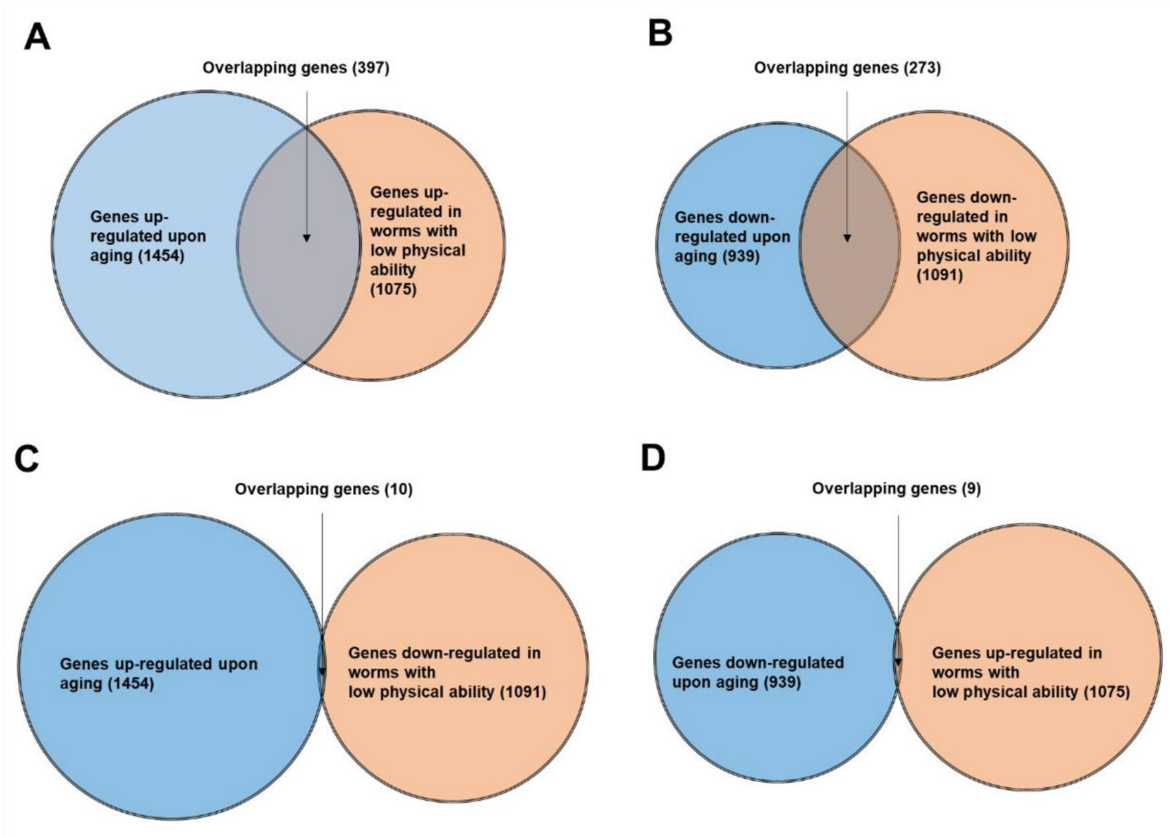

Supplementary Figure 2. Concordant changes in gene expression during aging and decreased MV. (**A**) Venn diagram depicting the overlap between genes that are up-regulated in worms with low physical ability and upon aging. (**B**) Venn diagram depicting the overlap between genes that are down-regulated in worms with low physical ability and upon aging. (**C**) Genes overlapping between genes up-regulated upon aging and down-regulated in low MV worms. (**D**) Genes overlapping between genes down-regulated upon aging and up-regulated in low MV worms.

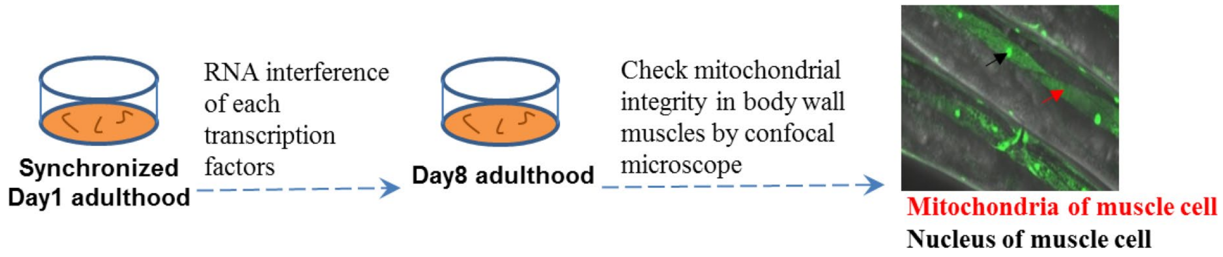

Supplementary Figure 3. Schematic diagram of RNA interference screen for age-associated mitochondrial defense genes. Synchronized day 1 of adulthood were transferred to each of transcription factors' RNAi plates, and their mitochondrial morphology in the body wall muscle were observed at day 8 of adulthood. Red and black arrows represent mitochondria and nucleus of body wall muscle cell, respectively.

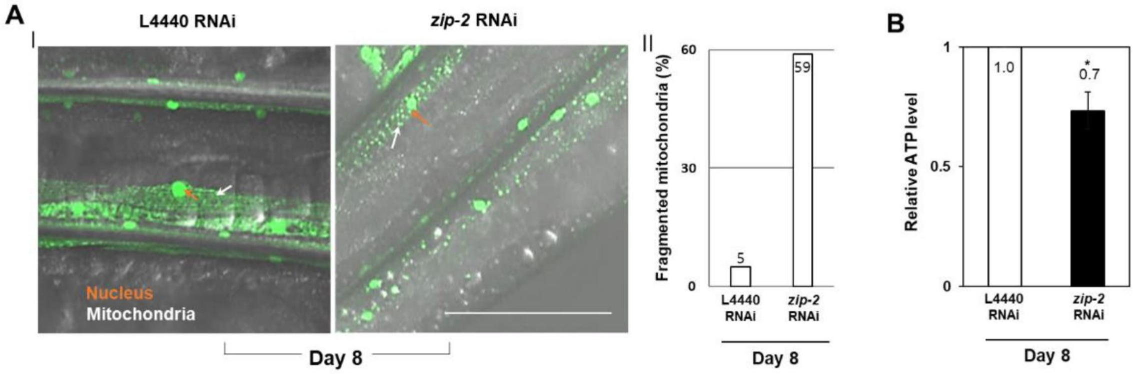

Supplementary Figure 4. ZIP-2 mitigates mitochondrial disintegration in aging. (**A**) (I) Representative images of mitochondrial morphologies in body wall muscle at day 8 of adulthood in L4440 RNAi (n=23) or *zip-2* RNAi (n=22) worms. The orange and white arrows indicate the nucleus and mitochondria of muscle cells, respectively. Scale bar: 100 µm. (II) Qualitative analysis of mitochondrial morphology observed at day 8 of adulthood in L4440 RNAi and *zip-2* RNAi worms. Bars represent the proportion of worms with fragmented mitochondria. (**B**) Relative ATP levels at day 8 of adulthood in L4440 RNAi and *zip-2* RNAi worms. The n value represents total number of tested worms by two independent experiments. Significance was determined using a two-tailed, unpaired *t*-test. * $P < 0.05$.

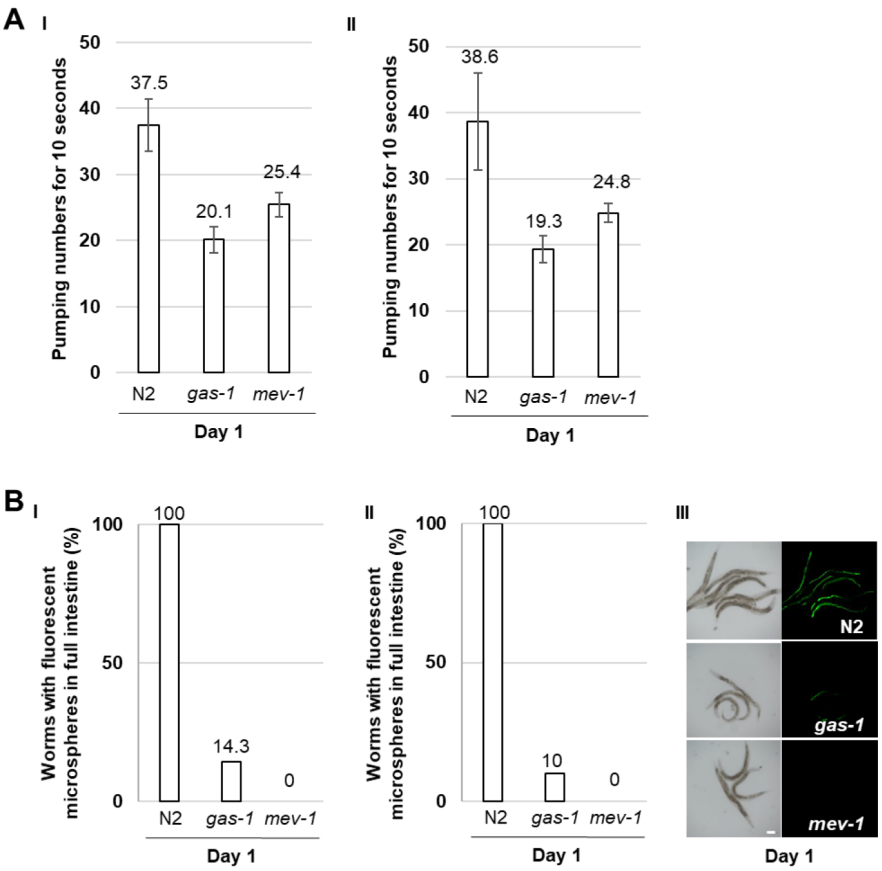

Supplementary Figure 5. Muscle functions of pharynx or intestine in wild-type or mitochondrial electron transport chain mutant strains. (**A**) Pharyngeal pumping rates of wild-type (N2) (n=43), *gas-1(fc21)* mutant worms (n=13), and *mev-1(kn-1)* mutant worms (n=13). Error bars represent *standard deviation (S.D.).* The n value represents total number of tested worms by two independent experiments. (**B**) (I and II) The proportion of worms with fluorescent microspheres in full intestine in wild-type (n=24), *gas-1(fc21)* mutant worms (n=24), and *mev-1(kn-1)* mutant worms (n=22). The n value represents total number of tested worms by two independent experiments. (III) Representative images of worms with accumulated microspheres of green fluorescence for 60 min at day 1 of adulthood. Scale bar: 100 µm.

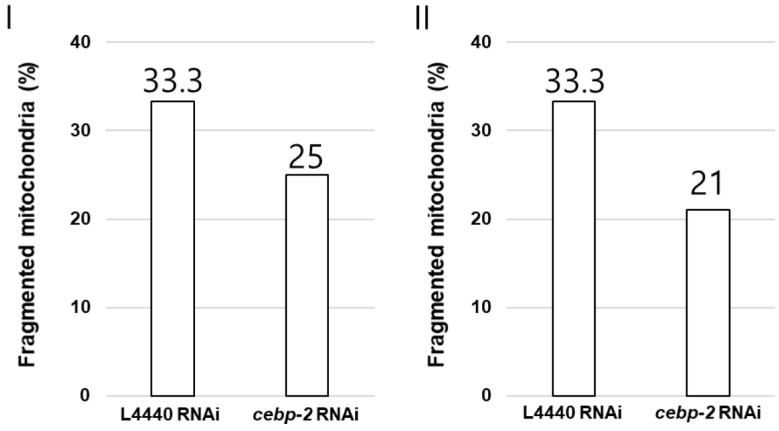

Supplementary Figure 6. The proportion of worms with fragmented mitochondria in L4440 or *cebp-2* RNAi conditions. Qualitative analysis of mitochondrial morphology observed at day 8 of adulthood in L4440 RNAi (n=24) and *zip-2* RNAi worms (n=35). Bars represent the proportion of worms with fragmented mitochondrial forms. The n value represents total number of tested worms by two independent experiments.
