# Supplementary material for: A cellular surveillance and defense system that delays aging phenotypes in C. elegans
Source: Aging (Albany NY). 2020 Apr 29;12(9):8202–20. doi: 10.18632/aging.103134 (PMC7244029; doi:10.18632/aging.103134)
Supplement: Supplementary Tables 2, 3 [file aging-12-103134-s001..pdf]

SUPPLEMENTARY TABLES

Please browse Full Text version to see the data of Supplementary Table 1.

Supplementary Table 1. (Uploaded xls file) DEGs in between worms with high MV and worms with low MV. Differentially expressed genes (DEGs) of each microarray data set were determined by three criteria (I) present probe; (II) combined p-values (<0.05); and (III) absolute log2 fold-changes (>0.58, 1.5 fold change).

Supplementary Table 2. Mitochondria-related genes up- and down-regulated in worms with low MV.

No.	WB ID	Sequence name	Gene name	UP/DOWN in low MV
1	WBGene00000182	B0336.2	*arf-1.2*	UP
2	WBGene00000230	F27C1.7	*atp-3*	UP
3	WBGene00018763	F53F10.1	*F53F10.1*	UP
4	WBGene00002981	ZK593.6	*lgg-2*	UP
5	WBGene00008980	F20D1.10	*tag-299*	UP
6	WBGene00016943	C55B7.4	*acdh-1*	DOWN
7	WBGene00001566	C28C12.9	*acdh-13*	DOWN
8	WBGene00000206	F35G12.10	*asb-1*	DOWN
9	WBGene00010015	F54B3.3	*atad-3*	DOWN
10	WBGene00010042	F54C9.6	*bcs-1*	DOWN
11	WBGene00011872	T20G5.10	*blos-1*	DOWN
12	WBGene00077500	C27H6.9	*C27H6.9*	DOWN
13	WBGene00014172	ZK970.2	*clpp-1*	DOWN
14	WBGene00000550	F55H2.6	*clu-1*	DOWN
15	WBGene00018240	F40G9.2	*cox-17*	DOWN
16	WBGene00000933	C14A4.2	*dap-3*	DOWN
17	WBGene00001028	F22B7.5	*dnj-10*	DOWN
18	WBGene00001093	T12E12.4	*drp-1*	DOWN
19	WBGene00017159	F01F1.2	*F01F1.2*	DOWN
20	WBGene00017301	F09F7.4	*F09F7.4*	DOWN
21	WBGene00017770	F25B4.7	*F25B4.7*	DOWN
22	WBGene00018426	F44E7.4	*F44E7.4*	DOWN
23	WBGene00019022	F58A6.1	*F58A6.1*	DOWN
24	WBGene00001486	F59G1.7	*frh-1*	DOWN
25	WBGene00011083	R07B1.12	*glo-1*	DOWN
26	WBGene00016266	C30F12.7	*idhg-2*	DOWN
27	WBGene00002879	C29E4.8	*let-754*	DOWN
28	WBGene00008415	D2030.5	*mce-1*	DOWN
29	WBGene00022373	Y92H12BR.8	*mrpl-15*	DOWN
30	WBGene00022470	Y119C1B.4	*mrpl-19*	DOWN
31	WBGene00012992	Y48C3A.10	*mrpl-20*	DOWN
32	WBGene00019076	F59A3.3	*mrpl-24*	DOWN
33	WBGene00020796	T25D3.2	*mrpl-28*	DOWN
34	WBGene00011334	T01E8.6	*mrps-14*	DOWN
35	WBGene00022583	ZC262.8	*mrps-18A*	DOWN
36	WBGene00016142	C26E6.6	*mrps-18C*	DOWN
37	WBGene00017319	F09G8.3	*mrps-9*	DOWN
38	WBGene00020625	T20F5.3	*mrrf-1*	DOWN
39	WBGene00009559	F39B2.11	*mtx-1*	DOWN
40	WBGene00004190	T27F6.5	*pars-2*	DOWN
41	WBGene00004092	F44C4.5	*ppt-1*	DOWN
42	WBGene00020022	R12C12.1	*R12C12.1*	DOWN
43	WBGene00011309	R186.8	*R186.8*	DOWN
44	WBGene00044305	F56H1.6	*rad-8*	DOWN
45	WBGene00015391	C03G5.1	*sdha-1*	DOWN

46	WBGene00044049	C41C4.10	*sfxn-5*	DOWN
47	WBGene00004932	C08A9.1	*sod-3*	DOWN
48	WBGene00016844	C50F7.4	*sucg-1*	DOWN
49	WBGene00011662	T09F3.2	*T09F3.2*	DOWN
50	WBGene00020636	T20H4.5	*T20H4.5*	DOWN
51	WBGene00006572	C06G3.11	*tin-9.1*	DOWN
52	WBGene00021248	Y22D7AL.10	*Y22D7AL.10*	DOWN
53	WBGene00012885	Y45F10D.4	*Y45F10D.4*	DOWN
54	WBGene00014227	ZK1128.1	*ZK1128.1*	DOWN
55	WBGene00022862	ZK1236.1	*ZK1236.1*	DOWN

Supplementary Table 3. 21 transcription factor genes in RNAi screening.

NO.	WB ID	Sequence name	Gene name	Fold change in low MV/high MV
1	WBGene00019327	K02F3.4	*zip-2*	3.4
2	WBGene00003602	H01A20.1	*nhr-3*	3.3
3	WBGene00009827	F47G4.6	*hmg-6*	3.3
4	WBGene00006775	F56A12.1	*unc-39*	3.2
5	WBGene00001210	K11G9.4	*egl-46*	3.2
6	WBGene00000430	C16C2.1	*ceh-5*	3.1
7	WBGene00000435	W03A3.1	*ceh-10*	3.0
8	WBGene00003653	C06C6.4	*nhr-63*	2.9
9	WBGene00000561	C34E10.7	*cnd-1*	2.9
10	WBGene00000463	C28A5.4	*ceh-43*	2.8
11	WBGene00003651	W01D2.2	*nhr-61*	2.6
12	WBGene00004949	K08A8.2	*sox-2*	2.6
13	WBGene00003648	R11G11.2	*nhr-58*	2.5
14	WBGene00004786	F44A6.2	*sex-1*	2.4
15	WBGene00001081	F47A4.2	*dpy-22*	2.3
16	WBGene00001976	T05A7.4	*hmg-11*	2.2
17	WBGene00019741	M02H5.3	*nhr-201*	2.1
18	WBGene00012317	W06H12.1	*ztf-6*	2.1
19	WBGene00009939	F52F12.6	*ztf-11*	2.1
20	WBGene00004751	K10G6.3	*sea-2*	2.0
21	WBGene00022554	ZC204.2	*duxl-1*	2.0
